# DISTRIBUTION, PREDICTORS, AND CLINICAL RELEVANCE OF 5-YEAR CHANGE IN FRAILTY MEASURES

**DOI:** 10.1093/geroni/igz038.2971

**Published:** 2019-11-08

**Authors:** Megan Huisingh-Scheetz, Kristen Wroblewski, Mark Ferguson, Elbert Huang, Linda Waite, L P Schumm

**Affiliations:** University of Chicago, Chicago, Illinois, United States

## Abstract

Implementing frailty assessment into routine clinical practice is a priority. Gait speed and performance on 5 repeated chair stands are two measures of frailty. We face a number of clinical implementation challenges: (1) We lack normative data for U.S. older adults and (2) The clinical relevance of change in frailty measures is unclear. The National Social Life, Health and Aging Project dataset allows an examination of the distribution of 3-meter gait and 5-repeated chair stands times as well as 5-year change in these measures in a nationally-representative, community-dwelling older adult sample. Dr. Huisingh-Scheetz will describe demographic predictors of change in these measures as well as determine whether baseline plus 5-year change in these measures predicts loss of independence in activities of daily living (ADLs).

